# 7^th^ Brazilian Guideline of Arterial Hypertension: Chapter 5 - Therapeutic Decision and Targets

**DOI:** 10.5935/abc.20160155

**Published:** 2016-09

**Authors:** MVB Malachias, AA Andrea Araujo Brandão, S Kaiser, O Moreira Filho

## Introduction

The therapeutic management of elevated BP includes non-pharmacological measures and the use of antihypertensive drugs to reduce BP, protect target organs and prevent CV and renal outcomes.^1-3^ Non-pharmacological measures have proven efficient to reduce BP, although limited by medium- and long-term lack of adherence to treatment. A systematic review^4^ of studies with a minimum duration of 12-24 months, combining dietary interventions and moderate-to-high-intensity physical activity in patients using or not medications, has revealed a reduction in SBP and DBP for < 12 months of -4.47 (-7.91 to -1.04) mm Hg and -1.10 (-2.39 to 0.19) mm Hg, respectively. For 12 to 24 months, the reductions were -2.29 (-3.81 to -0.76) mm Hg and -1.00 (-3.22 to 1.22) mm Hg in SBP and DBP, respectively. The direct impact of those measures on the risk of CV outcomes is uncertain, the studies are small and short, and the effects on other RF could contribute to CV protection.

On the other hand, results of randomized placebo-controlled clinical trials on the use of antihypertensive drugs for hypertensive individuals have clearly shown a significant reduction in CV mortality, stroke, MI and HF. It is worth noting that most of those studies have assessed individuals aged ≥ 55 years, at high CV risk and for a follow-up period of 3 to 6 years, hindering, thus, the extrapolation of those benefits for long-term treatment and patients with other characteristics.

The therapeutic decision should be based not only on BP levels, but consider the presence of RF, TOD and/or established CVD.

### Treatment decision making

#### Approach to stages 2 and 3 and/or high-risk hypertensives

Individuals with BP ≥ 160/100 mm Hg and/or high CV risk, even if stage 1, should begin immediately drug treatment associated with non-pharmacological therapy.^5-9^ Studies on antihypertensive drugs, most of which performed with patients with that profile, have shown efficacy in BP reduction and CV protection.^5-8^ Non-pharmacological therapy alone cannot reduce BP sufficiently to meet the recommended BP target,^4^ despite being an effective adjuvant treatment to control BP and other CVRF often present. Although the absolute benefit of therapy is higher in stages 2 and 3, it also increases the residual risk because of the frequent presence and influence of other RF and already installed TOD, neutralizing part of that benefit. This reinforces the importance of approaching CV risk globally.^7-9^

#### Approach to stage 1 hypertensives at low and intermediate risk

The last international guidelines^2,3^ point to a gap in the evidence favoring the impact of antihypertensive therapy on the outcome reduction of stage 1 hypertensives at low-to-intermediate risk. A meta-analysis^10^ of four randomized studies with a minimum duration of 1 year has included 8,912 individuals with SBP of 140-159 mm Hg and/or DBP of 90-99 mm Hg. As compared to placebo, the treatment for 5 years has not reduced total mortality, CAD, stroke or CV events, having even increased by five times the chance of adverse events. Another meta-analysis^6^ by the *Blood Pressure Lowering Treatment Trialists' Collaboration* , selecting ten randomized studies on treatment vs placebo for stage 1 hypertensives, has shown a reduction in the risk for stroke, total mortality and CVD, but had included individuals on antihypertensive therapy and/or individuals with DM. When such patients were excluded, the results lost statistical power. Later, the analysis of six studies on stage 1 SAH, involving 16,036 individuals, excluding those with DM and those on baseline antihypertensive therapy, has shown significant reductions in the risk of stroke (36%), CAD (12%), CV death (22%) and total mortality (18%). An analysis restricted to stage 1 SAH and low to intermediate risk of events (up to 5% in 10 years) has shown a reduction in the risk of those same outcomes, apparently strengthening those findings. However, the absolute benefit increased as the global CV risk increased.^7-9^

Recently, the HOPE-3 Study has contributed to that subject.^11^ In a significant population sample of 12,705 individuals at intermediate CV risk (38% hypertensive), the treatment combining candesartan and hydrochlorothiazide (16 mg/day and 12.5 mg/day, respectively) has shown a 27%-reduction in the risk of composite primary outcome (mortality, stroke and non-fatal AMI) in patients with initial SBP > 143.5 mm Hg (upper tertile). Those with lower SBP, in the first and second tertiles, however, showed no reduction in CV outcomes, and, on the contrary, the risk for the study's primary outcome tended to increase, although not significantly, in individuals of the first tertile of SBP.

The result of the HOPE-3 Study supports a recent meta-analysis on hypotensive therapy stratified by CV risk, in which a BP reduction of 4.6/3 mm Hg from baseline systolic levels around 155 mm Hg has determined an 18% reduction in the risk of outcomes.^12^

Thus, for stage 1 hypertensives at intermediate or low CV risk, non-pharmacological therapy should be attempted^13,14^ for 3 and 6 months, respectively (GR: I; LE: B), after which, the lack of BP control determines the beginning of pharmacological therapy. It is mandatory, however, to follow those individuals up with periodical assessment of adherence to the non-pharmacological measures. Once the lack of adherence or worsening of BP levels is detected, pharmacological therapy should be started. It is worth noting that the intervention in stage 1 hypertensives at low risk can prevent progression of the CV risk. Currently, the wide availability of antihypertensive drugs favors a safe and well-tolerated treatment.

#### Approach to BP levels of 130-139/85-89 mm Hg

Several meta-analyses with individuals with PH have shown a greater risk of progression to SAH and of CV events in that group after adjusting for other RF.^15-20^ Interventions in individuals with those BP levels are justified by the finding that half of the burden attributed to BP occurs in those with SBP between 130 and 150 mm Hg.^21^ It is worth noting, in that BP range, the expressive number of individuals with CVD, kidney disease, DM, metabolic syndrome and multiple CVRF.^21^ Non-pharmacological measures are recommended for that BP range. Prospective, observational studies of lifestyle intervention have shown lower risk of developing AH in those adopting a healthy lifestyle.^13,22-24^ (GR: I; LE: A).

Drug treatment can be considered for prehypertensive individuals with BP of 130-139/85-89 mm Hg and previous history of CVD^25^ (GR: IIb; LE: B) or individuals at high CV risk with no CVD^26^ (GR: IIb, LE: B), but there is no evidence of benefit for those at intermediate risk.^11^ Studies of renin-angiotensin-aldosterone system (RAAS) blockers for individuals with BP of 130-139/85-89 mm Hg at high CV risk have shown a reduction in the incidence of AH.^27,28^ There is no consistent evidence of the benefit of antihypertensive therapy to CV outcomes in that group. Thus, the decision to institute pharmacological therapy should be customized.

#### Approach to hypertensive elderly

The most common mechanism of AH in the elderly is wall stiffness of the large arteries, leading to a predominant increase in SBP, and maintenance or decrease in DBP. There is no study assessing the impact of antihypertensive therapy in this group with baseline SBP between 140 and 159 mm Hg. Because of the inclusion criteria of the major studies, the BP level at entrance in the study was ≥ 160 mm Hg, clearly showing the advantage of the intervention from that level onward. Lower thresholds have not been tested, leaving a gap of evidence. Presumably, the benefits demonstrated on TOD for the general population should not differ from those of the elderly population. Studies conducted with individuals aged ≥ 80 years have shown favorable results of the use of antihypertensive drugs for those with BP ≥ 160 mm Hg, especially to prevent stroke and HF.^29,30^ Thus, antihypertensive pharmacological therapy in the elderly should begin from SBP levels ≥ 140 mm Hg onward, as long as well tolerated and considering the individual's general conditions.^31^ (GR: IIb; LE: B).

In very old individuals (aged ≥ 80 years), the threshold to begin pharmacological therapy increases to SBP ≥ 160 mm Hg.^29,30^ (GR: I; LE: A).

#### Approach to youngsters with isolated systolic hypertension

The ISH is frequent among healthy male youngsters aged < 30 years and can be associated with normal central BP. In such cases, the treatment yields no significant benefits,^32^ and non-pharmacological measures should be adopted, with TOD monitoring. When managing ISH, pharmacological therapy should begin immediately if the CV risk is high. If DBP elevation occurs, the same criteria for the treatment of the general population should be adopted.

Tables 1 and 2 show the grades of recommendation and levels of evidence for beginning the treatment.

**Table 1:** Recommendations to begin antihypertensive therapy: lifestyle interventions and pharmacological therapy

Situation	Population (casual measure)	Recommendation	Class	Level of evidence
Beginning of lifestyle interventions	All hypertension stages and BP of 135-139/85-89 mm Hg	At the time of diagnosis	I	A
Beginning of pharmacological therapy	Stage 2 and 3 hypertensives	At the time of diagnosis	I	A
	Stage 1 hypertensives and high CV risk	At the time of diagnosis	I	B
	Elderly hypertensivesaged < 80 years	SBP ≥140 mmHg	IIa	B
	Elderly hypertensivesaged ≥ 80 years	SBP ≥160 mmHg	IIa	B
	Stage 1 hypertensives and intermediate or low CV risk	Wait 3-6 months for the effect of lifestyle interventions	IIa	B
	Individuals with BP of 130-139/85-89 mm Hg and preexisting CVD or high CV risk	At the time of diagnosis	IIb	B
	Individuals with BP of 130-139/85-89 mm Hg and no preexisting CVD and low or intermediate CV risk	Not recommended	III	-

BP: blood pressure; SBP: systolic blood pressure; CV: cardiovascular; CVD: cardiovascular disease.

**Table 2:** Blood pressure targets to be met according to individual characteristics

Category	Target recommended	Class	Level of evidence
Stage 1 and 2 hypertensives with intermediate or low CV risk and stage 3 AH	< 140/90 mm Hg	I	A
Stage 1 and 2 hypertensives with high CV risk	< 130/80 mm Hg*	I	A**

CV: cardiovascular; AH: arterial hypertension.

* For patients with CAD, BP should not be < 120/70 mm Hg, particularly those with DBP < 60 mm Hg, because of the risk of coronary hypoperfusion, myocardial damage and CV events.

** For diabetic patients, the class of recommendation is IIb, level of evidence B.

### BP targets

Recent international guidelines^2,3^ have recommended more conservative BP targets for the elderly and those at high CV risk, such as diabetic individuals, mainly because of the lack of evidence supporting recommendations for different types of patients. However, meta-analyses^7,9^ and the SPRINT Study^31^ have suggested reviewing those recommendations. A meta-analysis^7^ of 32 controlled and randomized studies with 104,359 individuals with different initial BP levels (stages 1 to 3) has compared the impact of the BP levels obtained (SBP: < 150 mm Hg, < 140 mm Hg and < 130 mm Hg; and DBP: < 90 mm Hg and < 80 mm Hg) on total and CV mortality and CV outcomes (stroke, CAD and HF). The BP reduction to 140-149 mm Hg (mean, 143.3 mm Hg) as compared to > 150 mm Hg has shown a significant decrease in the risk of total and CV mortality, stroke, CAD and HF. The comparison of the SBP levels obtained of 30-139 mm Hg (mean, 137.2 mm Hg) with values > 140 mm Hg has shown reductions in the risk of total and CV mortality, stroke, CAD, but not of HF. In addition, the comparison of the SBP levels achieved of 120-129 mm Hg (mean, 126.8 mm Hg) with those > 130 mm Hg has revealed a reduction in the risk of total mortality and stroke. The same analysis carried out for DBP revealed that DBP of 80-89 mm Hg (mean, 86.6 mm Hg) as compared to > 90 mm Hg reduced the risk of total and CV mortality, stroke, CAD and HF, while DBP of 70-79 mm Hg (mean, 78.5 mm Hg) as compared to > 80 mm Hg reduced the risk of only stroke. Thus, the BP target < 140/90 mm Hg has unequivocal benefits in reducing the risk of CV mortality and outcomes, and the BP target < 130/80 mm Hg is safe and provides more protection against stroke.

The randomized, controlled clinical trial SPRINT^31^ has included 9,361 individuals > 50 years, with SBP of 130-180 mm Hg and high CV risk (risk ≥ 15% within 10 years by the Framingham score, CVD, kidney disease or ≥ 75 years), excluding those with DM, polycystic kidney disease or previous stroke. The study population was randomized for more intense (< 120 mm Hg) and less intense (< 140 mmHg) SBP reduction. The composite primary outcome was the occurrence of AMI or other acute coronary syndrome, stroke, HF and CV death. In the first year, the BP levels achieved were 121.4/68.7 mm Hg and 136.2/76.3 mm Hg, respectively. The early interruption of the study in 3.26 years was due to the benefit demonstrated in the more intense SBP treatment arm, with a 25% reduction in the risk of the study's primary outcome as compared to that of the less intense SBP treatment arm (1.65%/year vs 2.19%/year, HR = 0.75; 95% confidence interval: 0.64-0.89; p < 0.001). In addition, the more intense treatment group had a 27% reduction (HR = 0.73; 95% confidence interval: 0.60-0.90; p = 0.003) in the risk of total mortality. The benefit was demonstrated in pre-specified subgroups. The incidence of adverse events, mainly hypotension, syncope, electrolyte disorders and acute kidney injury, was higher in the group with more intense BP reduction. In individuals ≥ 75 years, the occurrence of adverse events was similar to that in the entire population studied. Despite the greater rate of severe adverse events, the CV benefits and the benefits on mortality overlapped the risks of adverse events.

There is a major controversy regarding diabetic patients. The ACCORD study,^33^ including 4,733 diabetic patients, also randomized for SBP reduction < 120 mm Hg and < 140 mm Hg, could not reduce significantly the risk of the study's primary outcome (HR = 0.88; 95% confidence interval: 0.73-1.06; p = 0.20), and, thus, does not support the recommendations for stricter BP targets in that group of patients. In the ACCORD study,^33^ the SBP means achieved in the first year of treatment were 119.3 mm Hg and 133.5 mm Hg for the arms of greater and smaller SBP reductions, respectively. In addition, it is worth noting that, even with a small number of events, the more intense treatment arm reduced the risk of stroke by 41% (HR = 0.59; 95% confidence interval: 0.39-0.89; p = 0.01) and had a low incidence of adverse events.

Several differences in the conception of the SPRINT^31^ and ACCORD^33^ studies indicate the need for caution when interpreting their apparently different conclusions: the number of patients recruited in the ACCORD study was almost half that of the SPRINT study and with a lower mean age. The SPRINT study included older individuals (28% ≥ 75 years) and with CKD. The 2X2 factorial design of the ACCORD study, simultaneously assessing the effect of glycemic control, might have contributed to reduce the statistical power of the population sample, because, in later analyses, the sample restriction to individuals with strict BP control regardless of their serum glucose levels has revealed a 26% risk reduction, in accordance with the SPRINT data.^31^ Therefore, the findings suggest that the divergence in the results of the two studies could have been due to differences in study design, interactions between treatments, or to chance. However, specific changes in arteriolar function and blood flow in DM could have influenced the difference between the results of the two studies. Regarding DBP, the HOT study^34^ has shown, in diabetic patients, a 51% reduction in the risk of major CV outcomes in the treatment arm aimed at reaching DBP < 80 mm Hg (actual mean achieved: 81 mm Hg) as compared to the treatment arm aimed at reaching DBP < 90 mm Hg.

Based on the results of clinical trials and systematic reviews cited, this guideline chose to recommend a BP target lower than 130/80 mm Hg for patients at high CV risk. Exceptions apply to two situations: 1) for diabetic patients - so far considered as at high risk - that PB target was not supported by the results of the ACCORD study, therefore, the recommendation was defined as GR: IIb; LE: B; 2) for patients with CAD, recent records and cohort studies have shown an increase in fatal and non-fatal CV events,^35^ in addition to an increase in troponin^36^ when BP levels were < 120/70 mm Hg, especially DBP < 60 mm Hg.^36^ Thus, for those patients, BP target should be within a narrower safe range (< 130/80 mm Hg, but not < 120/70 mm Hg).

The BP target of stage 3 hypertensive individuals, despite their high CV risk, should be < 140/90 mm Hg,^7^ because there is no scientific evidence supporting greater BP reductions. (GR: I; LE: A).

For elderly hypertensives ≥ 80 years, there is no evidence of benefits deriving from BP levels < 140 mm Hg, in addition to the increased likelihood of adverse effects. The HYVET Study supports the recommendation of BP target < 150/90 mm Hg with a reduction in the risk for stroke and HF.^30^ The presence of ISH requires care regarding the excessive reduction in DBP, which should be maintained over 60 mm Hg or even over 65 mm Hg in the presence of CAD.^34^ (GR: IIb; LE: B). In the SPRINT Study, the elderly aged ≥ 75 years allocated to the more intense BP treatment arm (mean SBP achieved, 123.4 mm Hg) as compared to the group of standard SBP reduction (mean BP achieved, 134.8 mm Hg) had a 24% reduction in the risk of the study's primary outcome, regardless of the degree of fragility, and no increase in the number of adverse events in relation to the rest of the study population.^37^ Thus, the BP targets for the elderly should be defined in the same way they are for other adults; however, BP reduction should be performed carefully and considering the presence of comorbidities and the use of multiple medications.

Table 1 shows the major recommendations for BP targets.

Hypertensives without proper BP control should undergo monthly medical assessments, aimed at reaching the BP target recommended as soon as possible by using sequential therapeutic adjustments. Whenever possible, BP control should be confirmed with outside-the-office BP measurements, by use of either 24-hour ABPM or HBPM. In the elderly and those with significant BP elevations, BP levels should be reduced carefully and progressively, on a case-by-case basis, depending on the patient's general conditions, presence of comorbidities and of concomitant medication.
